# Food consumption and food exchange of caged honey bees using a radioactive labelled sugar solution

**DOI:** 10.1371/journal.pone.0174684

**Published:** 2017-03-29

**Authors:** Robert Brodschneider, Anika Libor, Vera Kupelwieser, Karl Crailsheim

**Affiliations:** Institute of Zoology, University of Graz, Graz, Austria; University of North Carolina at Greensboro, UNITED STATES

## Abstract

We measured the distribution of sugar solution within groups of caged honey bees (*Apis mellifera*) under standard *in vitro* laboratory conditions using ^14^C polyethylene glycol as a radioactive marker to analyze ingestion by individual bees after group feeding. We studied the impact of different experimental setups by varying the number of bees, age of bees, origin of bees, duration of experiment, the amount of available diet, and the influence of the neurotoxic pesticide imidacloprid in the diet on the feeding and food sharing behavior (trophallaxis). Sugar solution was non-uniformly distributed in bees in 36 out of 135 cages. As a measure of the extent to which the sugar diet was equally distributed between caged bees, we calculated the (inner 80%) intake ratio by dividing the intake of the 90^th^ percentile bee by the intake of the 10^th^ percentile bee. This intake ratio ranged from 1.3 to 94.8 in 133 individual cages, further supporting a non-uniform distribution of food among caged bees. We can expect a cage with 10 or 30 bees containing one bee that ingests, on average, the 8.8-fold of the bee in the same cage ingesting the smallest quantity of food. Inner 80% intake ratios were lower in experiments with a permanent or chronic offering of labelled sugar solution compared to temporary or acute feedings. After pooling the data of replicates to achieve a higher statistical power we compared different experimental setups. We found that uniform food distribution is best approached with 10 newly emerged bees per cage, which originate from a brood comb from a single colony. We also investigated the trophallaxis between caged honey bees which originally consumed the diet and newly added bees. Color marked bees were starved and added to the cages in a ratio of 10:5 or 20:20 after the initial set of bees consumed all the labelled sugar solution. The distribution of the labelled sugar solution by trophallaxis within 48 hours to added bees was 25% (10:5) or 45% (20:20) of the initial sugar solution. Imidacloprid at its median lethal dose (LD_50_) in the sugar solution reduced this post-feeding food transmission to 27% (20:20). Our results show that differences in food intake exist within caged bees that may lead to differential exposure that can influence the interpretation of toxicity tests.

## Introduction

Exposure to insecticidal pesticides is among other stress factors like parasitic mites, pathogens, poor hive management, loss of foraging habitat, and/or poor nutrition thought to be associated with declines in honey bee health or outright losses of honey bee colonies [1–8]. Effects of pesticides on honey bees are studied *in vivo* at the colony level in field or semi-field studies, and are studied *in vitro* using small groups of bees or individual bees under laboratory conditions [9, 10]. For oral toxicity testing, individual bees may be exposed to liquid diets by a single feeding method [11] or a number of caged bees is fed simultaneously by the group feeding method [10] which is the most commonly used method for experiments with pesticides and pathogens. The latter test assumes uniform distribution of food among caged honey bees and that trophallaxis plays an important role in distributing food and hence the test substance.

Trophallaxis (exchange of liquids between colony members) is a common and important interaction in social insects [12, 13]. The flow of nutrients among bees can be observed by the addition of a radioactive tracer to the food [14–16]. For example, six forager bees transferred ^32^P labelled sugar water to 62% of the foragers and 20% of the total worker population of a honey bee colony within 4 hours [17]. The age of bees involved in active and passive trophallaxis is of interest considering the age-related division of labor of honey bees. Free [18] observed in hive experiments that bees of all ages feed nest mates of all ages. He found a preference of bees feeding similar ages except for newly emerged bees and one day old bees, which received rather than provided food. Honey bees of one age class are found in same nest areas, increasing their probability for trophallactic interactions [13]. In contrast to colony experiments, Moritz and Hallmen [19] found in cage experiments that one day old bees and bees between 15 and 20 days old were most active donors; whereas, bees between 5 and 8 days old showed low trophallactic activity. Pershad [20] showed in cage experiments that 4 day old bees fed other bees more often than 8 day old bees. Reasons for the discrepancy in these results might be, besides the age of the recipients, caused by genetic relatedness to the donors and the filling of their honey stomachs [13], the group size [20] and disturbance in behavior caused by different experimental designs [13,10].

Besides data from behavioral observations, little is known about the efficiency of food distribution of caged honey bees. For example, infection with *Nosema spp*. has been found to be unequally distributed among honey bees after group feeding of a spore containing sugar solution [21]. Experiments with caged bees hence imply the risk that some bees consume more of a sugar solution diet with dissolved test substances or pathogen in it than others which could have implications on toxicity assessment in laboratory studies [22]. Standard methods and regulations for group feeding have so far not been evaluated for uniform food distribution [9, 23]. In this study, we therefore tested if honey bees distribute a sugar solution diet uniformly among caged individuals. In a series of experiments, we investigated the food consumption, trophallaxis, and the distribution of sugar solution among caged honey bees. We temporarily or permanently offered a sugar solution labelled with ^14^C polyethylene glycol (PEG) to a group of bees. In addition, we examined if the neonicotinoid pesticide imidacloprid (in acute and chronic testing conditions) has an impact on trophallaxis among caged honey bees, which could further affect exposure during *in vitro* toxicity studies. Moreover we measured the extent of trophallactic distribution of food solution in cages, by adding marked bees to cages after the original bees consumed all labelled sugar solution.

## Material and methods

Experiments were carried out from July to September 2014 using *A*. *mellifera carnica* from colonies of the institute of zoology. Since *A*. *mellifera* is not a protected species, no specific permission was required to perform the study. Experimental cages were modified 200 mL plastic cups [24]. Due to possible radioactive contaminations during the experiments, all cages were used once and then discarded. For different research questions, we used 10 to 40 bees per cage. Before each experiment, bees were starved up to two hours as specified in the OECD guidelines for the testing of chemicals No. 213 [23]. During starvation period dead bees were replaced before applying different experimental feeding regimes (temporary, permanent, acute or chronic) and caging conditions.

### Feeding regimes

For “temporary feeding” bees received a defined amount of ^14^C PEG sugar solution once at the start of the experiment, which was then followed by unlabelled food for maintenance. Under “permanent feeding” regimes, bees had *ad libitum* access to ^14^C PEG labelled sugar solution throughout the whole experiment. For “acute feeding” bees received 100 μL of a labelled sugar solution containing imidacloprid once at initial feeding followed by unlabelled food without imidacloprid for maintenance. During “chronic feeding” bees had *ad libitum* access to a labelled sugar solution with imidacloprid.

### Radioactive labelling

We used ^14^C labelled polyethylene glycol (^14^C PEG, stock solution: 1.85 MBq/mL diluted to 18.5 Bq/μL, Perkin Elmer) as a marker to detect food ingested by caged honey bees. ^14^C PEG was used since the compound is not absorbed by the intestinal tract nor is it metabolized [25, 26]. The radioactive marker was added to a 50% sucrose solution, a standard *in vitro* diet [10, 9]. We adjusted the activity of ^14^C PEG in the sugar solution diet depending on feeding regime and number of bees per cage. The activity for temporary and acute feeding regimes was 37.0 to 111.0 Bq per 100 μL sugar solution and for permanent and chronic feeding regimes we used 7.4 to 74.0 Bq per 100 μL sugar solution, according to the number of bees in the cage and duration of the experiment (S1 Table).

### Imidacloprid doses and concentrations

For the acute feeding regime, we used 4.5 ng/bee (LD_50_) analytical grade imidacloprid (Sigma Aldrich^®^) and 1760 μg/L (LC_50_) for chronic feeding [22]. For sublethal effects of imidacloprid on food sharing behavior we used 0.09 ng/bee (^1^/_50_ LD_50_) and 35.2 μg/L (^1^/_50_ LC_50_), respectively.

### Post-feeding food circulation

An initial set of bees (10 or 20 bees per cage) received temporary or acute feedings. After ten initial bees completely consumed the labelled sucrose solution we added five bees (marked on the thorax) and offered unlabelled sucrose solution in experiment TA12. In experiment TA13, 20 bees were added after 20 bees finished feeding on labelled sugar solution. In experiment AA4 the initial sucrose solution contained imidacloprid at 90 ng/100 μL (the LD_50_ dosage for 20 bees) in addition to the radioactive marker.

### Experiment procedure

Newly emerged worker bees (0–24 hours old) were collected randomly for 28 experiments. Therefore, combs containing sealed brood were removed from bee hives and stored in an incubator at 34.5°C [10]. To test the influence of genetic origin, newly emerged honey bees were taken either from a comb from a single colony or mixed from four different colonies with unrelated queens (experiments T7, T8, Table 1). For three experiments adult bees of mixed age were taken directly from comb surface of the opened hive as described in the OECD guidelines for the testing of chemicals No. 213 [23]. Again, bees originated from a single colony (T9, T10, Table 1) or were taken from 4 different colonies with unrelated queens and mixed (T11).

**Table 1:** Experimental outlines. Different experimental setups for temporary^1^ (T), acute^2^ (A), permanent^3^ (P), chronic^4^ (C) feeding regimes and post-feeding food circulation experiments (TA and AA) with varying number of bees per cage. Amount of ^14^C labelled sugar solution diet (μL), experiment duration (hours), age of bees (hours), genetic origin and number of replicates are shown.

Exp. setup	Feeding regime	sugar solution (μL)	Duration [h]	Age [h]	Genetics	Replicates
**10 bees per cage**						
T1	Temporary	100	3–7	0–24	Mix	6
T2	Discarded					
T3	Temporary	25	48	0–24	Mix	3
T4	Temporary	100	48	0–24	Mix	3
T5	Temporary	100	48	0–24	Mix	6
T6	Temporary	100	48	0–24	Mix	6
T7	Temporary	100	48	0–24	Single colony	6
T8*	Temporary	100	48	0–24	Mix from 4 colonies	5
T9	Temporary	100	48	Mixed age	Single colony	6
T10	Temporary	100	48	Mixed age	Single colony	3
T11	Temporary	100	48	Mixed age	Mix from 4 colonies	3
P1	Permanent	*Ad libitum*	48	0–24	Mix	6
P2	Permanent	*Ad libitum*	48	0–24	Mix	6
P3	Permanent	*Ad libitum*	48	0–24	Mix	6
P4	Permanent	*Ad libitum*	72	0–24	Mix	6
P5	Permanent	*Ad libitum*	72	0–24	Mix	6
A1	Acute LD_50_	100	3–7	0–24	Mix	6
A2	Acute LD_50_	100	48	0–24	Mix	6
A3	Acute ^1^/_50_ LD_50_	100	48	0–24	Mix	6
C1	Chronic LC_50_	*Ad libitum*	48	0–24	Mix	6
C2	Chronic LC_50_	*Ad libitum*	48	0–24	Mix	6
C3	Chronic LC_50_	*Ad libitum*	48	0–24	Mix	6
C4	Chronic ^1^/_50_ LC_50_	*Ad libitum*	48	0–24	Mix	6
**30 bees per cage**						
T14	Temporary	100	3–7	0–24	Mix	3
T15	Temporary	100	48	0–24	Mix	3
P6	Permanent	*Ad libitum*	48	0–24	Mix	3
A5	Acute LD_50_	100	48	0–24	Mix	3
C5	Chronic LC_50_	*Ad libitum*	48	0–24	Mix	1
C6	Chronic LC_50_	*Ad libitum*	48	0–24	Mix	3
**Addition of bees**						
TA12	Temporary	100	48	0–24	Mix	5
TA13	Temporary	100	48	0–24	Mix	2
AA4	Acute LD_50_	100	48	0–24	Mix	2
					Replicates total:	144

*12 bees per cage

^1^ temporary: test bees initially provided with defined amount (25μL/100μL) of ^14^C labelled diet, followed by unlabelled diet *ad libitum* for maintenance

^2^ acute: test bees initially provided with defined amount (25μL/100μL) of ^14^C labelled diet containing LD_50_ or ^1^/_50_ LD_50_ imidacloprid, followed by unlabelled diet *ad libitum* for maintenance

^3^ permanent: test bees provided with ^14^C labelled diet *ad libitum*

^4^ chronic: test bees provided with ^14^C labelled diet containing LD_50_ or ^1^/_50_ LD_50_ imidacloprid *ad libitum*

Each experiment started when labelled sugar solution diet was offered to the caged bees. Due to different research questions, the duration of the experiments varied. The majority of the experiments lasted 48 hours, but we stopped three experiments immediately after the initial feeding solution was consumed (Table 1, T1, A1, and T14). Two experiments under permanent feeding regime lasted 72 hours (Table 1, P4, P5). Bee mortality was recorded as soon as the initial sugar solution was entirely consumed and at 24, 48 and 72 hours respectively. Dead bees were removed and stored in the freezer at -20°C for further analyses.

### Sample preparation

Each bee was homogenized individually in 1 mL of 80% ethanol by sonification (Branson Sonifier^®^ Cell Distruptor B15) according to Crailsheim [27]. Bee samples were extracted for 20 minutes at 60°C (Koltermann) and centrifuged at 15.000 rpm (10.000 g) for 5 minutes (BHG Hermle Z 230 M). Radioactivity was quantified in a 500 μL aliquot of the supernatant using liquid scintillation counter (Packard Tri-Carb 1900 CA). All samples were cooled for 12 hours in the dark after adding 10 mL scintillation cocktail (Carl Roth; Rotiszint^®^ eco plus) prior to analyses in the liquid scintillation counter. The obtained decays per minute (dpm) were multiplied by factor 2.217 to correct for the weight of the total sample. This factor was determined as follows: mean weight of honey bee (91.6 mg, N = 20) plus mean weight of 1 mL ethanol (840.8 mg, N = 20) results in 932.4 mg containing 100% of the radioactivity. Consequently, the aliquot of 500 μL ethanol contained 45.1% radioactivity of the original sample volume.

Additional wash and swipe samples from all used cages and feeder tube surfaces were taken to determine ^14^C activity of spilled sugar solution from possible feeder movement by bees. The wash and swipe samples were additional information to calculate the ^14^C recovery. Due to *ad libitum* feedings in permanent and chronic feeding regimes, the remaining sugar solution was added to 10 mL scintillation cocktail cooled for 12 hours and analyzed in the fluid scintillation counter.

### Data analysis

In a first step, we created standard curves to convert weight corrected dpm data (hereafter referred to as raw (dpm)) into μL of labelled food and then corrected for recovery for each experiment (S1 File). Raw (dpm), uncorrected (μL) and corrected data (μL) were significantly correlated (Spearman-Correlation test, p ≤ 0.01, S2 Table). Therefore we used the data corrected for recovery for all further analysis.

We tested the intake of sugar solution of bees per cage on assumption of normality with the Shapiro Wilk test [28]. Two-sample Kolmogorov-Smirnov tests were used to test for uniform distribution of intake of sugar solution and to compare intake distributions of bees from different experimental setups. For a uniform distribution we assumed that n bees in a cage consumed ^1^/_n_ parts of sugar solution each. The two-sample Kolmogorov-Smirnov statistics quantifies the greatest deviation (D) of two distributions and assigns a critical value (CV). For example, CVs, as used in our experiment are: 0.30, 0.17, 0.12 and 0.10 for N = 10, 30, 60 and 90 bees per testing group [29]. If the observed D is greater than or equal to the CV, then the two distributions are considered significantly different at p < 0.05. For an increase of statistical power we pooled data of all replicates within one experimental setup.

We calculated the full range intake (ingested diet) ratio by dividing the maximum intake by the minimum intake per bee in one cage (intake ratio min-max). We further trimmed the data to the inner 80% to rule out extreme fluctuations by dividing 90^th^ percentile intake by the 10^th^ percentile intake (inner 80% intake ratio).

We used the Kruskal-Wallis test for comparison between donor bees and added bees in post feeding sugar solution circulation experiments (TA12, TA13 and AA4, Table 1). As we found no significant differences in the intake of sugar solution within replicates (Kruskal-Wallis test, p > 0.05), we pooled data of donor bees and added bees of the two to five different replicates from one experimental setup. For comparison of food intake of bees that died prior to the end of the experiment versus bees surviving the experiments, data from all replicates of an experiment were pooled due to small numbers of dead bees. The intake of sugar solution between donor bees and added bees and between bees which died during experiments versus surviving bees per cage was analyzed using Mann-Whitney U-test. Statistical analyses using Kruskal-Wallis test, Mann-Whitney U-test and Spearman-Correlation test were performed with the statistic software SPSS^®^ (version 20). The two-sample Kolmogorov-Smirnov test was carried out manually in Microsoft^®^ Excel 2010.

## Results

### Recovery and correction of results

The mean recovery of ^14^C PEG (found in all bees, swipe and wash samples) for different experimental setups is shown in Table 2. On average 77.6% of the applied ^14^C was recovered in our experiments.

**Table 2:** Recovery of ^14^C PEG for the different applied feeding regimes. Mean recovery of ^14^C labelled sugar solution diet and number of replicates for experimental setups with 10 or 30 bees for temporary^1^ (T) and permanent^2^ (P) feeding regimes without imidacloprid and for acute^3^ (A) and chronic^4^ (C) feeding regimes with imidacloprid.

Feeding regime	Experiment	Mean Recovery in%	Replicates (cages)
**10 bees per cage**			
Temporary	T1, T3-T11, TA12, TA13	71.9	54
Acute	A1, A2, A3, AA4	72.6	20
Permanent	P1-P5	84.5	30
Chronic	C1-C4	89.9	24
**30 bees per cage**			
Temporary	T14, T15	78.9	6
Acute	A5	67.9	3
Permanent	P6	80.7	3
Chronic	C5, C6	74.5	4
Total		77.6	144

^1^ temporary: test bees initially provided with defined amount (25μL/100μL) of ^14^C labelled diet, followed by unlabelled diet *ad libitum* for maintenance

^2^ acute: test bees initially provided with defined amount (25μL/100μL) of ^14^C labelled diet containing LD_50_ or ^1^/_50_ LD_50_ imidacloprid, followed by unlabelled diet *ad libitum* for maintenance

^3^ permanent: test bees provided with ^14^C labelled diet *ad libitum*

^4^ chronic: test bees provided with ^14^C labelled diet containing LD_50_ or ^1^/_50_ LD_50_ imidacloprid *ad libitum*

### Uniform and normal distribution

In 99 (73.3%) of 135 individual cages there was a uniform distribution of food among caged bees while there was a non-uniform distribution in 36 (26.7%) (Table 3, TA12, TA13 and AA4 are excluded from this analysis, because of added bees). In cages with 30 bees in only 4 (25.0%) out of 16 cages food was shared uniformly compared to 79.8% in cages with 10 bees. All pooled replicates per experiment showed that there is a significant deviation from the uniform distribution (Kolmogorov-Smirnov test, p < 0.05). In one experiment (T7) with newly emerged bees originating from one colony, the value of the deviation from the theoretical uniform distribution was equal to the critical value (Kolmogorov-Smirnov test, p = 0.05). The distribution of food followed a normal distribution in 27 (20%) of cages.

**Table 3:** Abundance of cages with a uniform and normal distribution of a labelled sugar solution diet for all replicates for temporary^1^ (T) and permanent^2^ (P) feeding regimes without imidacloprid and for acute^3^ (A) and chronic^4^ (C) feeding regimes with imidacloprid. Mean and range (min-max) for full range intake ratio and inner 80% intake ratio are shown for each experiment.

Experiment	Uniform distribution	Normal distribution	Mean intake ratio (range)	Mean inner 80% intake ratio (range)
**10 bees per cage**				
T1	6/6	2/6	20.8 (3.6–45.0)	4.3 (1.9–10.3)
T3	2/3	1/3	16.5 (8.3–24.8)	8.4 (7.1–10.6)
T4	3/3	0/3	7.7 (2.1–14.5)	5.8 (1.8–10.4)
T5	4/6	0/6	19.0 (8.7–30.8)	7.8 (1.5–13.2)
T6	6/6	0/6	15.8 (3.5–40)	3.7 (1.9–7.2)
T7	6/6	0/6	3.1 (2.0–4.1)	2.1 (1.3–3.0)
T8*	3/5*	1/5*	70.9 (2.7–310.1)*	8.6 (1.7–25.1)*
T9	2/6	1/6	65.5 (3.7–158.6)	30.4 (2.2–94.8)
T10	2/3	0/3	25.7 (2.6–68.6)	15.6 (2.5–39.5)
T11	3/3	0/3	17.8 (12.6–23.5)	9.5 (3.8–22.6)
P1	5/6	1/6	9.7 (4.6–14.8)	5.1 (2.7–8.9)
P2	6/6	0/6	39.6 (2.41–191.05)	2.9 (1.7–5.1)
P3	4/6	1/6	8.3 (2.8–23.1)	16.8 (1.5–77.0)
P4	6/6	0/6	31.9 (3.0–117.3)	2.6 (2.2–3.3)
P5	6/6	0/6	47.7 (7.8–128.6)	8.1 (1.8–28.2)
A1	1/6	2/6	108.9 (44.7–245.9)	27.4 (3.5–51.1)
A2	5/6	1/6	10.1 (3.1–24.7)	6.2 (2.6–12.8)
A3	5/6	0/6	78.5 (4.5–302.5)	16.4 (2.8–64.7)
C1	6/6	2/6	3.9 (2.6–5.0)	2.0 (1.6–2.6)
C2	5/6	3/6	11.9 (2.9–28.8)	3.0 (1.7–6.3)
C3	3/6	0/6	14.8 (6.1–25.0)	7.7 (3.2–18.5)
C4	6/6	2/6	3.9 (2.8–6.0)	2.4 (1.7–3.9)
TOTAL	95/119	17/119	$\bar{\text{X}}$ = 29.5	$\bar{\text{X}}$ = 8.8
**30 bees per cage**				
T14	0/3	3/3	116.5 (42.9–190.1)	23.7 (4.7–41.0)
T15	0/3	2/3	26.9 (13.4–39.7)	6.0 (4.7–8.6)
P6	2/3	0/3	11.3 (9.9–12.7)	2.7 (2.1–3.0)
A5	0/3	2/3	36.4 (13.4–77.1)	9.4 (4.7–17.3)
C5	1/1	0/1	17.4**	5.6**
C6	1/3	3/3	18.3 (8.9–33.6)	3.4 (3.0–4.0)
TOTAL	4/16	10/16	$\bar{\text{X}}$ = 35.5	$\bar{\text{X}}$ = 8.8

*12 bees per cage;

**only one replicate

^1^ temporary: test bees initially provided with defined amount (25μL/100μL) of ^14^C labelled diet, followed by unlabelled diet *ad libitum* for maintenance

^2^ acute: test bees initially provided with defined amount (25μL/100μL) of ^14^C labelled diet containing LD_50_ or ^1^/_50_ LD_50_ imidacloprid, followed by unlabelled diet *ad libitum* for maintenance

^3^ permanent: test bees provided with ^14^C labelled diet *ad libitum*

^4^ chronic: test bees provided with ^14^C labelled diet containing LD_50_ or ^1^/_50_ LD_50_ imidacloprid *ad libitum*

### Intake ratio

**Fig 1:**
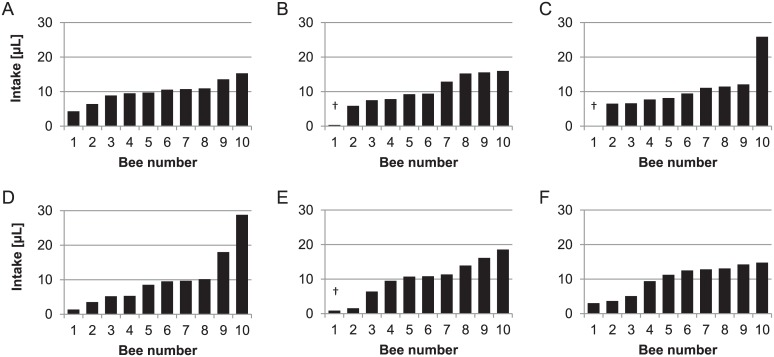
Individual intake per caged bee for six replicates (A-F) from experiment T1. Intake immediately after bees finished temporary feeding on 100 μl of ^14^C-labelled sugar diet. Full range intake ratio is the highest quantity of diet ingested by a bee divided by the lowest quantity of diet ingested by a bee. Inner 80% intake ratio likewise is second highest quantity ingested divided by second lowest quantity ingested. The inner 80% intake ratios in this experiment for replicates A-F are: 2.1, 2.6, 1.9, 5.2, 10.3 and 3.9, respectively. † indicates bees that died during the feeding phase.

**Fig 2:**
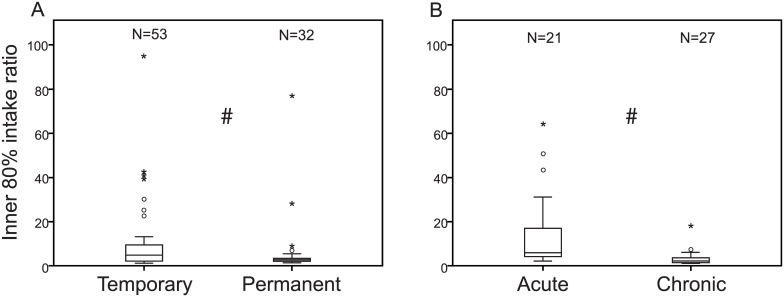
Inner 80% intake ratios of caged honey bees from temporary, permanent, acute and chronic feeding regimes. **A**. Inner 80% intake ratios (ratio of second highest quantity ingested to that of the second lowest quantity ingested) for pooled data from all replicates of temporary^1^ and permanent^3^ feeding of ^14^C-labelled sugar solution. **B**. Inner 80% intake ratios for pooled data from all replicates of acute^2^ and chronic^4^ feeding of ^14^C-labelled sugar solution spiked with imidacloprid. # indicates differences at p < 0.05, Mann-Whitney U-test. ° indicates outliers (values between 1.5 and 3 times the interquartile range) and * indicates far outliers (more than 3 times the interquartile range). ^1^ temporary: test bees initially provided with defined amount (25μL/100μL) of ^14^C labelled diet, followed by unlabelled diet *ad libitum* for maintenance ^2^ acute: test bees initially provided with defined amount (25μL/100μL) of ^14^C labelled diet containing LD_50_ or ^1^/_50_ LD_50_ imidacloprid, followed by unlabelled diet *ad libitum* for maintenance ^3^ permanent: test bees provided with ^14^C labelled diet *ad libitum* ^4^ chronic: test bees provided with ^14^C labelled diet containing LD_50_ or ^1^/_50_ LD_50_ imidacloprid *ad libitum*

The individual intake per caged bee is shown for six replicates of experiment T1 in Fig 1. The mean full range intake ratio per experiment (as determined by the maximum divided by the minimum intake) ranged from 3.1 (T7, Table 3) to 116.5 (T14), the inner 80% intake ratio from 2.0 (C1) to 30.4 (T9). The intake ratio trimmed to the inner 80% is naturally lower than the untrimmed ratio, except in experiment P3 in which we excluded one cage because of an invalid division by zero. Note that mean intake ratios per experimental setup are reported, although the minimum and maximum range is also presented, to illustrate that in single cages full range intake ratios of up to 310.1 have been detected (see T8, Table 3). The lowest inner 80% intake ratio found in a cage was 1.3 (one replicate of T7), whereas the highest was 94.8 (T9, Table 3). Temporary feedings resulted in statistically higher intake ratios than permanent feedings, the same applies for acute feedings compared to chronic feedings (Fig 2, Mann-Whitney U-test, p <0.05). There was no effect of imidacloprid detected on trimmed intake ratio for temporary versus acute feeding or permanent versus chronic experiments (Fig 2, Mann-Whitney U-test, p > 0.05). Inner 80% intake ratios of cages with ten or 30 bees did not differ (Table 3, Mann-Whitney U-test, p > 0.05).

### Comparison of food distributions

**Fig 3:**
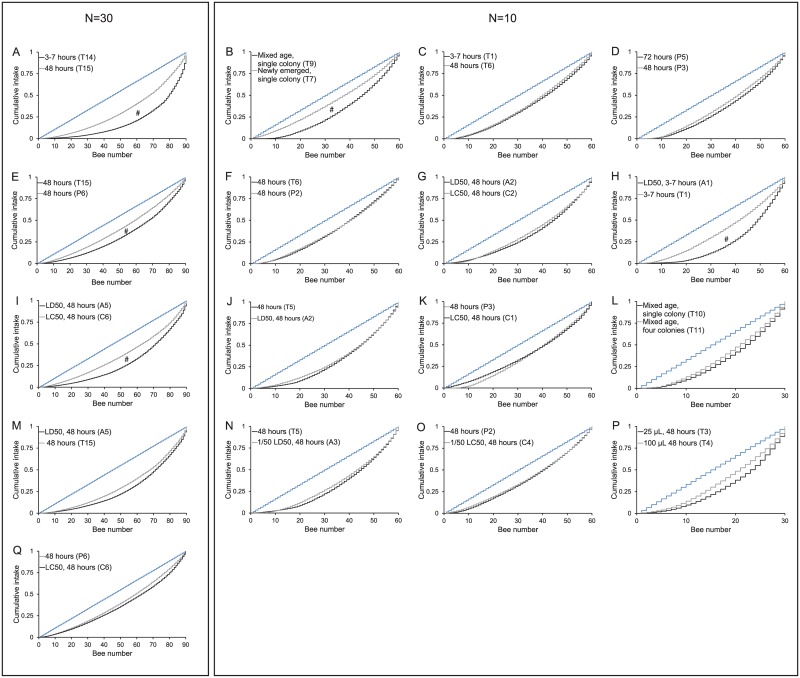
Comparison of cumulative intake of sugar diet between different experimental setups for temporary^1^ (T) and permanent^2^ (P) feeding regimes without imidacloprid and for acute^3^ (A) and chronic^4^ (C) feeding regimes with imidacloprid. Pooled data from replicates of the two different experimental setups are shown (grey and black) referring to an assumed theoretical uniform distribution (light blue). Left column shows all experimental setups with N = 30 bees per cage, right column shows all experimental setups with N = 10 bees per cage. # indicates statistically different distributions of food between experimental setups. All experimental setups were statistically different from theoretical uniform distribution. (Kolmogorov-Smirnov test p < 0.05). ^1^ temporary: test bees initially provided with defined amount (25μL/100μL) of ^14^C labelled diet, followed by unlabelled diet *ad libitum* for maintenance ^2^ acute: test bees initially provided with defined amount (25μL/100μL) of ^14^C labelled diet containing LD_50_ or ^1^/_50_ LD_50_ imidacloprid, followed by unlabelled diet *ad libitum* for maintenance ^3^ permanent: test bees provided with ^14^C labelled diet *ad libitum* ^4^ chronic: test bees provided with ^14^C labelled diet containing LD_50_ or ^1^/_50_ LD_50_ imidacloprid *ad libitum*

We compared the distribution of ^14^C-labelled sugar solution of pooled experimental setups to identify effects of caging conditions on food distribution. All comparisons are shown in Fig 3, where the theoretical uniform distribution (every bee has ingested the same amount of food) can be seen as a diagonal stair function (blue line). The measured food distributions of two complementary experimental setups are shown as black and grey line, respectively. All functions eventually peak at the complete cumulative intake of all bees of an experiment (value of one). Experimental setups deviate from the uniform distribution pattern because bees with low numbers lag behind in consumption, whereas the bees with higher numbers have disproportionately ingested more. The closer the food distribution lines of experimental setups are to the diagonal stair function, the closer it is to a uniform distribution, which could be tested statistically.

#### Group composition

Newly emerged bees from a single colony significantly better distributed sugar solution than bees of undefined mixed ages taken from a single colony (Fig 3B, Kolmogorov-Smirnov test, p < 0.05). Both experimental setups did not reach theoretical uniform distribution (Kolmogorov-Smirnov test, p < 0.05) though. Bees originating from one colony did not share sugar solution better than a mixture of bees from four different colonies regardless of age (Fig 3L, Kolmogorov-Smirnov test, p > 0.05). Neither experimental setup reached theoretical uniform distributions (Kolmogorov-Smirnov test, p < 0.05).

#### Caging condition

In 48 hours ten caged bees did not share their sugar diet more uniformly than immediately after temporary feeding, which lasted between three and seven hours (Fig 3C, Kolmogorov-Smirnov test, p > 0.05). In contrast, thirty bees in a cage distributed labelled sugar diet more uniformly after 48 hours compared to the distribution immediately after temporary feeding (Fig 3A, Kolmogorov-Smirnov test, p < 0.05). Caged bees did not better share a permanent fed labelled sugar diet during 72 hours compared to the distribution after 48 hours (Fig 3D, Kolmogorov-Smirnov test, p > 0.05). Experimental setups did not reach theoretical uniform distribution (Kolmogorov-Smirnov test, p < 0.05).

Temporary and permanent feeding of ten caged bees did not differ statistically in food distribution after 48 hours (Fig 3F, Kolmogorov-Smirnov test, p > 0.05). Neither feeding regimes reached theoretical uniform distribution (Kolmogorov-Smirnov test, p <0.05). In contrast, 30 bees per cage better distributed sugar solution under permanent feeding conditions compared to temporary feeding (Fig 3E, Kolmogorov-Smirnov test, p < 0.05). Temporary and permanent feeding regimes however, did not reach theoretical uniform distribution (Kolmogorov-Smirnov test, p < 0.05).

#### Feeding volume

Ten caged bees did not better distribute a labelled sugar solution when offered temporary 100 μL or 25 μL (Fig 3P, Kolmogorov-Smirnov test, p > 0.05). Both experimental setups did not reach theoretical uniform distribution after 48 hours (Kolmogorov-Smirnov test, p < 0.05).

#### Effect of imidacloprid

Similar to what was observed in temporary and permanent feeding of sugar solution, there was no statistical difference in food distribution of ten caged bees in acute versus chronic feeding of sugar solution spiked with imidacloprid (Fig 3G, Kolmogorov-Smirnov test, p > 0.05). Again, both feeding regimes differed from theoretical uniform distribution (Kolmogorov-Smirnov test, p > 0.05). In contrast, 30 bees per cage distributed sugar solution better under chronic feeding conditions than in acute feeding after 48 hours (Fig 3I, Kolmogorov-Smirnov test, p < 0.05). However, neither acute nor chronic feeding regimes reached theoretical uniform distribution (Kolmogorov-Smirnov test, p < 0.05).

Immediately after initial feeding, temporary feeding resulted in a more uniformly distributed sugar solution than acute feeding containing LD_50_ imidacloprid in cages with 10 bees (Fig 3H, Kolmogorov-Smirnov test, p < 0.05). This effect cannot be found after 48 hours, regardless of whether cages contained 10 or 30 bees (Fig 3J and 3M, Kolmogorov-Smirnov test, p > 0.05). Again, theoretical equal distributions were not reached (Kolmogorov-Smirnov test, p < 0.05).

There was no statistically significant effect detected from exposure to imidacloprid on food distribution in permanent versus chronic feedings with LC_50_ imidacloprid in cages with 10 or 30 bees (Fig 3K and 3Q, Kolmogorov-Smirnov test, p > 0.05). Theoretical uniform distributions were not reached in either case (Kolmogorov-Smirnov test, p < 0.05). Likewise, there were no statistically significant differences in food distribution after 48 hours in temporary versus acute or permanent versus chronic feedings in experiments with the applied ^1^/_50_ LD_50_ or ^1^/_50_ LC_50_ of imidacloprid (Fig 3N and 3O, Kolmogorov-Smirnov test, p > 0.05). No distribution reached the theoretical uniform distribution (Kolmogorov-Smirnov test, p < 0.05).

### Mortality rate and radioactive label in dead and surviving bees

Mortality occurred in all feeding regimes except for experiments T3, T4, P2, T14 and P6 (Table 4). In acute and chronic toxicity tests, where 50% of the bees were expected to die, only the acute experiment A2 approached the 50% lethality level with 55% of the bees dead. In all the other toxicity tests, involving 50% lethality level or ^1^/_50_ of it, the mortality level was less than 26%. There were no significant differences in the sugar solution intake between bees which died during the study and those that survived in the acute experiments A2 and A5 and in the chronic experiments C1 and C3 (Table 4, Mann-Whitney U-test, p > 0.05). In all the other experiments, bees which died before the end of the study had ingested significantly less sugar solution diet than surviving bees in experiments with and without imidacloprid (Table 4, Mann-Whitney U-test, p < 0.05).

**Table 4:** Mortality rate and cumulative intake in dead and surviving bees in different experimental setups for temporary^1^ (T), acute^2^ (A), permanent^3^ (P) and chronic^4^ (C) feeding regimes. Imidacloprid, duration of experiments, bee mortality, median food intake of dead and surviving bees and the respective p-value for the comparison of median intake of dead and surviving bees (Mann-Whitney U-test) for all experiments are shown. For number of replicates see Table 1.

Experiment	Imidacloprid	Duration	Bee mortality (%)	Median food intake per bee (μL)		
				dead	surviving	p-value
**10 bees per cage**						
T1	-	3–7	5.0	0.9	9.7	0.00
T3	-	48	0	-	2.2	-
T4	-	48	0	-	10.6	-
T5	-	48	16.7	2.0	10.9	0.00
T6	-	48	5	0.4	10.0	0.00
T7	-	48	1.7	-	9.7	-
T8*	-	48	13.3	0.8	9.0	0.00
T9	-	48	16.7	0.4	10.9	0.00
T10	-	48	6.7	0.5	10.4	0.00
T11	-	48	6.7	0.9	11.0	0.00
P1	-	48	11.7	9.2	44.7	0.00
P2	-	48	0	-	37.9	-
P3	-	48	6.7	0	30.8	0.00
P4	-	72	1.7	-	68.0	-
P5	-	72	6.7	1.7	57.5	0.00
A1	LD_50_	3–7	25.0	0.6	10.4	0.00
A2	LD_50_	48	55.0	7.1	10.1	0.29
A3	^1^/_50_ LD_50_	48	6.7	0.3	9.6	0.00
C1	LC_50_	48	18.3	15.9	19.1	0.17
C2	LC_50_	48	25.0	5.1	20.7	0.00
C3	LC_50_	48	20.0	9.7	16.0	0.06
C4	^1^/_50_ LC_50_	48	1.7	-	21.9	-
**30 bees per cage**						
T14	-	3–7	0	-	1.5	-
T15	-	48	16.7	1.5	3.1	0.00
P6	-	48	0	-	28.9	-
A5	LD_50_	48	12.2	1.6	2.6	0.17
C5	LC_50_	48	5.6	6.0	19.4	0.00
C6	LC_50_	48	23.3	14.2	21.4	0.00

*12 bees per cage

^1^ temporary: test bees initially provided with defined amount (25μL/100μL) of ^14^C labelled diet, followed by unlabelled diet *ad libitum* for maintenance

^2^ acute: test bees initially provided with defined amount (25μL/100μL) of ^14^C labelled diet containing LD_50_ or ^1^/_50_ LD_50_ imidacloprid, followed by unlabellied diet *ad libitum* for maintenance

^3^ permanent: test bees provided with ^14^C labelled diet *ad libitum*

^4^ chronic: test bees provided with ^14^C labelled diet containing LD_50_ or ^1^/_50_ LD_50_ imidacloprid *ad libitum*

### Post-feeding circulation

**Fig 4:**
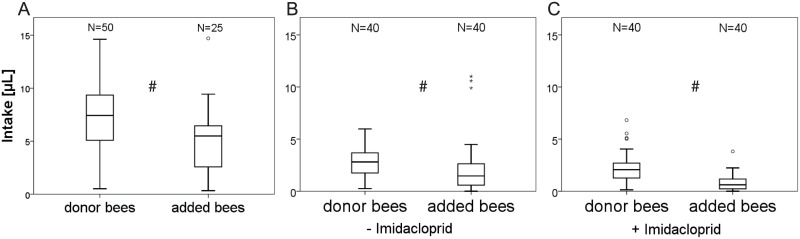
Sugar solution intake of caged bees (donor bees) fed with ^14^C-labelled sugar solution and bees added to cages after initial feeding phase (added bees) with and without imidacloprid. **A**: Intake of donor bees feeding on 100μL temporary ^14^C-labelled sugar solution and subsequent added bees at a ratio of 10:5 (pooled data from 5 replicates). **B**: Intake of donor bees feeding on 100μL temporary ^14^C-labelled sugar solution and subsequent added bees at a ratio of 20:20 (pooled data from 2 replicates). **C**: Intake of donor bees feeding on 100μL acute ^14^C-labelled sugar solution (LD_50_, 90ng/100μL) and subsequent added bees at a ratio of 20:20 (pooled data from 2 replicates). # indicates differences at p < 0.05, Mann-Whitney U-test. ° indicates outliers (values between 1.5 and 3 times the interquartile range) and *indicates far outliers (more than 3 times the interquartile range).

In the experiment TA12, 10 feeding bees plus 5 bees added afterwards, 25.0% of the initial fed sugar solution was detected in the added bees after 48 hours; in TA13 (20 + 20 bees) 45.1% was detected in the added bees (Table 5). In the experiment AA4 imidacloprid reduced the distribution of the initial fed sugar solution to the 20 added bees to 27.1% (mean of two replicates, Table 5). In all three experiments there was significantly less labelled sugar solution in added bees than in donor bees (Fig 4, Mann-Whitney U-test, p < 0.05).

**Table 5:** Food circulation among initially fed bees and added bees. Remaining and transmitted ^14^C-labelled sugar solution for individual cages and pooled per replicate for temporary (TA12 and TA13) and acute (AA4) feeding regimes with and without imidacloprid.

Number of original + added bees (experimental number)	Treatment	Cage	Remaining sugar solution in initial set of bees [%]	Transmitted sugar solution to added bees [%]
10 + 5 (TA12)	No imidacloprid	1	77.0	23.0
10 + 5 (TA12)	No imidacloprid	2	72.4	27.6
10 + 5 (TA12)	No imidacloprid	3	88.5	11.5
10 + 5 (TA12)	No imidacloprid	4	62.7	37.3
10 + 5 (TA12)	No imidacloprid	5	74.8	25.2
10 + 5 (TA12)	No imidacloprid	1–5 (pooled)	75.0	25.0
20 + 20 (TA 13)	No imidacloprid	1	55.6	44.4
20 + 20 (TA 13)	No imidacloprid	2	54.3	45.7
20 + 20 (TA 13)	No imidacloprid	1 + 2 (pooled)	54.9	45.1
20 + 20 (AA 4)	*imidacloprid* LD_50_	1	80.1	19.9
20 + 20 (AA 4)	*imidacloprid* LD_50_	2	65.6	34.4
20 + 20 (AA 4)	*Imidacloprid* LD_50_	1 + 2 (pooled)	72.9	27.1

## Discussion

We evaluated the suitability of honey bee group feeding to assess toxicity and toward a better understanding of group feeding under *in vitro* conditions. We used a stable radioactive marker (^14^C-labelled PEG) to trace food sharing among caged honey bees since polyethylene glycol accumulates in the body and therefore reflects the cumulative consumption of a caged bee [25, 26]. Toxicological studies are based on the assumption that caged honey bees all consume the same share of an offered food; however high variability in toxicity thresholds generated in laboratory-based studies and discrepancies between field-realistic levels suggest that exposure may not be adequately assessed [22]. These findings can be explained by either different sensitivities to test substances or simply by an unequal consumption [30, 31]. For the first time we quantified two characteristics of food distribution in caged honey bees. First, we established an intake ratio, which describes the gap between the bee ingesting the highest amount of food and the bee ingesting the lowest quantity of food in the cage. Second we tested the distribution of food in caged bees for a uniform distribution and compared different experimental setups.

Our results demonstrate that a uniform distribution (i.e. every bee in a cage consumes the same share of a fed sugar solution, for example 10 μL per bee when 100 μl food solution is offered) applied in 73% of 135 individually examined cages. The food sharing followed a normal distribution only in 20% of cases (Table 3). Analysis of pooled data indicated that the distribution of sugar solution never reached an uniform distribution in all experimental setups, a fact which is more apparent when comparing the individual intake of each bee. The individual intake of bees from six cages mimicking a setup similar to acute toxicity testing, but not containing a toxic substance is illustrated in Fig 1. The full range intake ratio, including all bees from a cage, is greatly affected by a single bee in the cage ingesting either very little or very much. We therefore trimmed this value to a more robust inner 80% intake ratio. We found a mean inner 80% intake ratio of 8.8 calculated from 133 cages that allowed this calculation, which means that on average one bee per cage is consuming 8.8-times more of an offered food solution than another bee in the same cage (Table 3). This already indicates that a uniform distribution of sugar solution among caged honey bees cannot be assumed (Table 3). In the 133 investigated single cages, the trimmed intake ratio ranged between 1.3 (which points to an almost perfect uniform distribution) to 94.8, which indicates a highly skewed distribution. In some cages, the full range intake ratio suggests an even more than 300-fold gap between the bee with the lowest and highest quantity of diet ingested. These imbalances in food distribution might partly explain that groups of bees fed diets with *Nosema* spores exhibited an uneven dispersal of infections among workers [21]. The intake ratios of different replicates as shown in Table 3 are summarized in Fig 2 to illustrate differences between temporary and permanent feeding conditions. While the intake ratio was not affected by imidacloprid, the intake ratio was higher in cages with temporary or acute feeding conditions compared to cages with chronic or permanent feedings, respectively. In temporary and acute feeding regimes probably few bees feed directly on the initially applied sugar solution. Other bees may receive some labelled food by trophallaxis and feed on the unlabelled food for maintenance provided after initial feeding phase. The longer exposure period in permanent or chronic feeding regimes hence led generally to lower differences in ingestion of the offered food. Intake ratios were not affected by the number of bees, ten or 30, in the cage (Table 3).

For cage experiments it is recommended to use worker bees from as many and diverse colonies as possible since bees of various ecotypes, populations, strains, or even single colonies of the same subspecies can differ in physiological, behavioral and biochemical traits [9, 10]. In our study, there was no difference in food distribution using mixed age bees from only one colony or a mixture of bees from four colonies (Fig 3L), which is remarkable because worker bees can discriminate between related and unrelated nest mates and are more prone to feed related bees [32–34]. We detected a significant improvement in food distribution for cages with newly emerged bees compared to cages with mixed age bees (Fig 3B). Based on our study results, we recommend newly emerged bees from the same colony to improve the food distribution in cages used for acute toxicity studies.

In our experiments, we explored the use of 10 or 30 bees per single cage [23, 35, 36] and found more often a uniform distribution of sugar solution in cages with 10 bees (Table 3). This might be due to the fact that each bee has an increased chance to feed directly on an offered sugar solution in smaller groups than in groups of 30 bees or more. In cages with 10 bees neither the amount of applied sugar solution nor the time given bees for trophallactic exchange (immediately after consummation, 48 hours or even longer) significantly improved the food distribution (Fig 3C, 3D and 3P). In contrast, in cages with 30 bees the food distribution was significantly affected by time and feeding regime. Our results suggest that food in larger groups with 30 bees per cage is more likely distributed via trophallactic contacts rather than directly ingesting test solution from the feeder (Fig 3A). Sugar solution is also more evenly distributed in permanent or chronic feeding than in temporary or acute feeding regimes (Fig 3E and 3I). This better distribution with time or pending on feeding regime was not apparent in experiments with 10 bees per cage (Fig 3C, 3F and 3G). Even bees feeding permanently on sugar solution differ in their individual intake, probably because of variable needs due to different activity in the cage.

We exposed caged bees to an oral LD_50_ of 4.9 ng imidacloprid per bee [22] to test for an influence that a pesticide itself could have on food sharing behavior. In some cases we observed signs of intoxication including loss of coordination and hyperactivity [31, 37]. These behavioral effects may have prevented caged bees from further feeding on the sugar solution, which significantly affected the dispersal of sugar solution immediately after consumption in cages with 10 bees (Fig 3H). Although imidacloprid does not bind permanently to the nicotinic acetylcholine receptor, effects may appear immediately after acute feeding and lead to visible signs of intoxication which may include impacts on feeding behavior [22, 30, 38–40]. Immediately after initial feeding phase, food was less evenly distributed in bees receiving imidacloprid compared to bees that received a pesticide free sugar solution. After 48 hours, the distribution pattern in bees treated with imidacloprid was not different to that from pesticide free controls anymore, both still not reaching uniform distributions. One possible explanation for this non-uniform distribution is that after caged bees consumed the acute sugar solution containing imidacloprid, they received untreated sugar solution *ad libitum* for maintenance, which may have diluted the pesticide-containing sugar solution in the honey stomach. Therefore, acute toxicity tests may only detect a small portion of adverse effects of a pesticide in honey bees due to short exposure duration of one to three days [41]. The pesticide may not have a lethal effect on the honey bee immediately after the initial feeding phase of the experiment, but mortality may occur later during the trial [42].

We found dead bees in all feeding regimes except for three temporary and two permanent experiments. In acute and chronic feeding regimes with LD_50_ and LC_50_ of imidacloprid, respectively, one acute experiment reached the expected bee mortality (Table 4, A2). For all other tests with imidacloprid, mortality was less than 26%. This is in some cases due to the short duration of experiments (bees in A1 for example were also exposed to LD_50_, but only for the time bees needed to consume the acute feeding) in other cases only a fiftieth of LD_50_ or LC_50_ was applied (A3 or C4).

In all experiments without pesticide (see temporary and permanent experiments in Table 4) bees that died in the course of the experiment consumed significantly less food than surviving bees, indicating that they either died early in the experiment or because of starvation. The results for experiments including the pesticide imidacloprid in LD_50_, LC_50_ or a fiftieth of it are not so clear. In none of the experiments had bees which died early ingested significantly more imidacloprid-spiked sugar solution than the surviving bees. In two acute (A2, A5) and two chronic (C1, C3) feeding scenarios dead bees ingested the same amount of food than surviving bees (Table 4). The experimental design of these experiments can be compared to classic toxicity tests, they lasted 48 hours and bees received LD_50_ or LC_50_, respectively. One possibility is that dead bees in these experiments died after consuming large quantities in a short period during the experiment, compared to surviving bees consuming the same quantity over a longer duration of time, i.e., 48 hours. Another possibility is that dead bees were more sensitive to imidacloprid than surviving bees [22, 43]. The isolation of grouped honey bees in cages has also been associated with an increased sensitivity to pesticides [44]. An alternative explanation is that bees simply die from other reason than pesticide. For example in one trial, 25% of bees were dead already after initial acute feeding and these bees had consumed significantly less sugar solution than surviving bees (Table 4). This is an example, for how toxicity testing might be misinterpreted, because one would generally assume that these bees died from the effects of pesticide. More detailed research using our labelling technique to study individual variability of sensitivity to a pesticide is recommended.

Post-feeding distribution (“circulation”) of food solution via trophallaxis is thought to play a major role in sharing toxic compounds both in the colony as well as in cages [9, 20]. In experiments where we added marked bees, these did never directly consume the labelled sugar solution served temporary, but after 48 hours contained a significant amount of the radioactive marker, similar to other experiments [13, 16, 17]. Nevertheless, we found more labelled food in donor bees than in the added bees. After 48 hours, about 25% of the temporary applied food in cages with ten original and five added bees can be found in the five added bees, whereas it is 45% in cages with a 20:20 ratio. The actual food distributions are slightly below to what one would expect from the ratio of bees and added bees. In experiments with imidacloprid at close to the LD_50_ the trophallactic distribution on the other hand was reduced almost by half (Table 5). Added bees maybe preferred consuming unlabelled sugar solution over being fed a pesticide solution by trophallactic contacts. Imidacloprid might have negatively influenced the trophallaxis among caged honey bees similar to what was observed in a study with the organophosphate pesticide coumaphos in which treated caged donor bees provided recipients with a food solution through a metal screen [45]. In the study with coumaphos mortality resulted from insufficient food transmission via impaired trophallactic contacts; this effect increased with coumaphos dose.

The effectiveness of trophallactic contacts is influenced by the physiological situation (*e*.*g*., extent to which the honey stomach is filled), the experimental setup, or even the receptiveness of other bees to trophallaxis [13]. While Korst and Velthuis [46] also consider the smell of the available diet to influence trophallactic contacts in honey bees, Kessler *et al*. [40] observed that bees cannot “taste” a pesticide and even suggested that honey bees preferred sugar solutions spiked with imidacloprid. These results contradict the findings of Desmedt *et al*. [47] who showed that caged bees preferred the pure untreated sucrose solution over a noxious solution as long as the bees were offered a food alternative. The absence of such an alternative diet led again to consumption of the noxious food solutions.

Although we confirmed that substantial amounts of an applied sugar solution circulate between caged bees via trophallaxis, our results indicate that caged honey bees do not share food equally. Our findings support the opinion of Blacquiére *et al*. [48] about extrapolating laboratory findings to field studies. Toxicity exposure models often assume a uniform exposure to pesticides. With respect to the complex environment in which honey bees forage and the social interactions within a honey bee colony, Sponsler and Johnson [49] point to a more sophisticated modelling of differential exposure and susceptibility of different members of a honey bee colony. The uneven distribution of food in cages reflects a different exposure, although it might not be comparable to the situation in a honey bee colony. In acute toxicity studies lasting only few days as suggested by some standardized test methods [23, 35, 36] uneven food distribution, as demonstrated in our study may be problematic. We could not identify any group or caging condition that drastically improved food sharing of caged honey bees, and found that even the testing compound itself could affect food distribution. The use of newly emerged and closely related honey bees appears to result in a more even distribution of food in cage experiments. Better food distribution can be expected during chronic conditions compared to acute feedings, especially when higher numbers of bees are used [9]. Another option for improving the food distribution might be to expose honey bees to a pesticide up to ten days as discussed by Decourtye & Devillers [41] or to feed honey bees individually [11, 21, 49]. Our results suggest further research is needed on the reliability of laboratory experiments toward ensuring a more uniform distribution of food among caged honey bees. Besides cage experiments, more natural settings for tests should be developed.

## Supporting information

S1 File Conversion of dpm to consumed volume. This includes text and one reference about 1. the standard curve, created for each experiment to convert the measured dpm into consumed μL of the applied sugar solution diet, 2. correction of the data with recovery factors, 3. correction of results for temporary and acute experiments and 4. correction of results for permanent and chronic experiments.
(DOCX)

S1 Table Composition of applied feeding solutions diets for temporary^1^, acute^2^, permanent^3^ and chronic^4^ feeding regimes. Feeding regime, experiment, the amount of applied sugar solution diet (μL), PEG (μL) and imidacloprid (μL) dose are shown. PEG (Perkin Elmer): 18.5 Bq/μL, Imidacloprid: LD_50_: 4.5 ng/bee, LC_50_:1760 μg/L, ^1^/_50_ LD_50_: 0.09 ng/bee, ^1^/_50_ LC_50_: 35.2 μg/L.
(DOCX)

S2 Table Correlations (Spearman rs) between raw data (dpm), uncorrected intake (μL) and corrected intake (μL) in temporary^1^, acute^2^, permanent^3^ and chronic^4^ experiments. Spearman’s rho (ρ) = correlation coefficient. * p≤0.01.
(DOCX)
