# Supplementary material for: Food consumption and food exchange of caged honey bees using a radioactive labelled sugar solution
Source: PLoS One. 2017 Mar 29;12(3):e0174684. doi: 10.1371/journal.pone.0174684 (PMC5371368; doi:10.1371/journal.pone.0174684)
Supplement: S1 File — This includes text and one reference about 1. the standard curve, created for each experiment to convert the measured dpm into consumed μL of the applied sugar solution diet, 2. correction of the data with recovery factors, 3. correction of results for temporary and acute experiments and 4. correction of results for permanent and chronic experiments. (DOCX) [file pone.0174684.s001.docx]

# File S1: Conversion of dpm to consumed volume.

# 1. Standard curve

A standard curve for each experiment was created to convert the measured dpm into µL. Therefore, three aliquots of different volumes were taken from the ^14^C labelled sugar solution, pipetted into a scintillation vial containing 10 mL scintillation cocktail and measured with the liquid scintillation counter as described above. The regression line allows converting dpm from scintillation measurements to µL using the following formula:$\mu L intake (x)=\frac{Found dpm (y)}{k}$.

# 2. Correction of the data with recovery factors

If the recovery is not 100%, a correction can be made with a recovery factor, applied to the data [1]. First, the found dpm in bees were converted into µL using the former formula. The µL intake by all bees in a cage, the swipe and wash sample of the cage and the feeder were summated to determine how much percent of the applied labelled sugar solution was recovered. The recovery rate is the fraction of the recovered radioactivity divided by the applied radioactivity, multiplied by 100 to give the percentage recovery:

$Recovery rate \%=\frac{Radioactivity recovered}{Radioactivity applied} x 100$.

The subsequent summation of found radioactive label always resulted in values lower than the applied radioactivity (=100%). Therefore a compensation for the actual found recovery was made. To calculate the values for a 100% recovery rate the recovered µL were corrected by multiplying them with a recovery correction factor. The correction factor was defined differently in temporary and acute experiments than in permanent and chronic experiments (see below). The corrected values were used for further statistical analyses.

$$Correction factor=\frac{Radioactivity applied (\mu L in feeder)}{Radioactivity recovered (\mu L)}$$

Whereby *‘Radioactivity recovered’* was defined as the sugar solution intake of all bees per cage (in µL) for temporary and acute feeding, excluding the wash and swipe sample. For permanent and chronic feeding, *‘Radioactivity recovered’* was defined as the intake of all bees per cage plus the wash and the swipe sample (in µL).

Correction factor for temporary and acute:

$$\left( 1. \right) Correction factor=\frac{Radioactivity applied (\mu L in feeder)}{Radioactivity recovered (intake in \mu L)}$$

(2.) Correction factor for permanent and chronic:

$$\left( 2. \right) Correction factor=\frac{Radioactivity applied (\mu L in feeder)}{Radioactivity recovered (intake+w+s in \mu L)}$$

where *w* stands for wash sample and *s* stands for swipe sample.

# 3. Correction of results for temporary and acute experiments

The recovery rates had to be standardized for each replicate to compare different experiments and replicates. Values were corrected in the way that the total sugar solution intake of all bees was 100 µL (or 25 µL in experiment T3). In doing so, swipe and wash samples were ignored. Therefore the real (uncorrected) intake was corrected with a correction factor for a recovery of 100% for all experiments containing temporary and acute sugar solutions. The corrected intake for 25 or 100 µL of labelled sugar solution was calculated with the following formula:

(1.) Correction factor for temporary /acute

$$\boldsymbol{=}\left( \mathbf{1.} \right)\boldsymbol{Correction factor \times intake per bee (\mu L)}$$

# 4. Correction of results for permanent and chronic experiments

The recovery rate was corrected to 100% for the purpose of experiment comparisons. Since the inserted 2000 µL, which should have been regained in the bees, wash sample and swipe sample, could not be found completely, the regained amount of µL was corrected to 2000 µL. In contrast to temporary feeding, the correction included the wash and the swipe sample, because bees were fed *ad libitum* and therefore they could not consume the whole labelled sugar solution.

(2.) Correction factor for permanent /chronic:

$\boldsymbol{=}\left( \mathbf{2}\mathbf{.} \right) \mathbf{Correction} \mathbf{factor}\boldsymbol{\times}\mathbf{intake} \mathbf{per} \mathbf{bee}\boldsymbol{(\mu}\mathbf{L}\mathbf{)}$

# Reference List:

1. Thompson M, Ellison SL, Fajgelj A, Willetts P, & Wood R. Harmonized guidelines for the use of recovery information in analytical measurement. Pure Appl Chem. 1999; 71(2): 337-348. doi.org/10.1351/pac199971020337
