# Supplementary material for: Food consumption and food exchange of caged honey bees using a radioactive labelled sugar solution
Source: PLoS One. 2017 Mar 29;12(3):e0174684. doi: 10.1371/journal.pone.0174684 (PMC5371368; doi:10.1371/journal.pone.0174684)
Supplement: S1 Table — Feeding regime, experiment, the amount of applied sugar solution diet (μL), PEG (μL) and imidacloprid (μL) dose are shown. PEG (Perkin Elmer): 18.5 Bq/μL, Imidacloprid: LD50: 4.5 ng/bee, LC50:1760 μg/L, 1/50 LD50: 0.09 ng/bee, 1/50 LC50: 35.2 μg/L. (DOCX) [file pone.0174684.s002.docx]

**Table S1: Composition of applied feeding solutions diets for temporary^1^, acute^2^, permanent^3^ and chronic^4^ feeding regimes.** Feeding regime, experiment, the amount of applied sugar solution diet (µL), PEG (µL) and imidacloprid (µL) dose are shown. PEG (Perkin Elmer): 18.5 Bq/µL, Imidacloprid: LD_50_: 4.5 ng/bee, LC_50_:1760 µg/L, ^1^/_50_ LD_50_: 0.09 ng/bee, ^1^/_50_ LC_50_: 35.2 µg/L.

| Feeding regime | Experiment | µL sugar solution (total) | µL PEG | µL imidacloprid | |
| --- | --- | --- | --- | --- | --- |
| **10 bees per cage** |  |  |  |  | |
| Temporary feeding | T1, T3-T6,T8*-T11 | 100 | 2 | | -- |
| Temporary feeding | T7 | 100 | 4 | -- | |
| Acute feeding (LD_50_) | A1,A2 | 100 | 2 | 2 | |
| Acute feeding (^1^/_50_ LD_50_) | A3 | 100 | 2 | 2 | |
| Permanent feeding | P1,P2,P4 | 1000 | 4 | -- | |
| Permanent feeding | P3,P5 | 1000 | 8 | -- | |
| Chronic feeding (LC_50_) | C1,C2,C3 | 1000 | 20 | 20 | |
| Chronic feeding (^1^/_50_ LC_50_) | C4 | 1000 | 20 | 20 | |
| **30 bees per cage** |  |  |  |  | |
| Temporary feeding | T14,T15 | 100 | 6 | | -- |
| Acute feeding (LD_50_) | A5 | 100 | 6 | 6 | |
| Permanent feeding | P6 | 1000 | 20 | -- | |
| Chronic feeding (LC_50_) | C5 | 1000 | 20 | 20 | |
| Chronic feeding (LC_50_) | C6 | 1000 | 40 | 20 | |
| **Adding bees experiment** *20 bees per cage* |  |  |  |  | |
| Acute feeding (LD_50_) | AA4 | 100 | 4 | 4 | |
|  |  |  |  |  | |
|  |  |  |  |  | |

*12 bees per cage;

^1^ temporary: test bees initially provided with defined amount (25µL/100µL) of ^14^C labelled diet, followed by unlabeled diet *ad libitum* for maintenance

^2^ acute: test bees initially provided with defined amount (25µL/100µL) of ^14^C labelled diet containing LD_50_ or ^1^/_50_ LD_50_ imidacloprid, followed by unlabelled diet *ad libitum* for maintenance

^3^ permanent: test bees provided with ^14^C labelled diet *ad libitum*

^4^ chronic: test bees provided with ^14^C labelled diet containing LD_50_ or ^1^/_50_ LD_50_ imidacloprid *ad libitum*
