# Supplementary material for: Food consumption and food exchange of caged honey bees using a radioactive labelled sugar solution
Source: PLoS One. 2017 Mar 29;12(3):e0174684. doi: 10.1371/journal.pone.0174684 (PMC5371368; doi:10.1371/journal.pone.0174684)
Supplement: S2 Table — Spearman’s rho (ρ) = correlation coefficient. * p≤0.01. (DOCX) [file pone.0174684.s003.docx]

**Table S2: Correlations (Spearman) between raw data (dpm), uncorrected intake (µL) and corrected intake (µL) in temporary^1^, acute^2^, permanent^3^ and chronic^4^ experiments.** Spearman’s rho (ρ) =correlation coefficient. * p≤0.01.

| Feeding regime | Raw data (dpm) vs. uncorrected intake (µL) | Raw data (dpm) vs. corrected intake (µL) | Uncorrected intake (µL) vs. corrected intake (µL) |
| --- | --- | --- | --- |
| Temporary | ρ=0.83* | ρ=0.74* | ρ=0.96* |
| Acute | ρ=0.83* | ρ=0.80* | ρ=0.99* |
| Permanent | ρ=0.35* | ρ=0.39* | ρ=0.99* |
| Chronic | ρ=0.42* | ρ=0.52* | ρ=0.98* |

^1^ temporary: test bees initially provided with defined amount (25µL/100µL) of ^14^C labelled diet, followed by unlabelled diet *ad libitum* for maintenance

^2^ acute: test bees initially provided with defined amount (25µL/100µL) of ^14^C labelled diet containing LD_50_ or ^1^/_50_ LD_50_ imidacloprid, followed by unlabelled diet *ad libitum* for maintenance

^3^ permanent: test bees provided with ^14^C labelled diet *ad libitum*

^4^ chronic: test bees provided with ^14^C labelled diet containing LD_50_ or ^1^/_50_ LD_50_ imidacloprid *ad libitum*
